# The Porosity Design and Deformation Behavior Analysis of Additively Manufactured Bone Scaffolds through Finite Element Modelling and Mechanical Property Investigations

**DOI:** 10.3390/jfb14100496

**Published:** 2023-10-08

**Authors:** Shummaila Rasheed, Waqas Akbar Lughmani, Muhammad Mahabat Khan, Dermot Brabazon, Muhannad Ahmed Obeidi, Inam Ul Ahad

**Affiliations:** 1 Department of Mechanical Engineering, Capital University of Science and Technology, Islamabad 44000, Pakistan; shummaila@cust.edu.pk (S.R.); drmahabat@cust.edu.pk (M.M.K.); 2 Faculty of Mechanical Engineering, Ghulam Ishaq Khan Institute of Engineering Sciences and Technology, Topi 23460, Pakistan; waqas.akbar@giki.edu.pk; 3 I-Form, The SFI Research Centre for Advanced Manufacturing, School of Mechanical and Manufacturing Engineering, Dublin City University, 09 Dublin, Ireland; dermot.brabazon@dcu.ie (D.B.); muhannad.ahmedobeidi@dcu.ie (M.A.O.)

**Keywords:** polymeric bone scaffolds, 3D printing, mechanical response, finite element method, deformation pattern, crushable foam plasticity model

## Abstract

Additively manufactured synthetic bone scaffolds have emerged as promising candidates for the replacement and regeneration of damaged and diseased bones. By employing optimal pore architecture, including pore morphology, sizes, and porosities, 3D-printed scaffolds can closely mimic the mechanical properties of natural bone and withstand external loads. This study aims to investigate the deformation pattern exhibited by polymeric bone scaffolds fabricated using the PolyJet (PJ) 3D printing technique. Cubic and hexagonal closed-packed uniform scaffolds with porosities of 30%, 50%, and 70% are utilized in finite element (FE) models. The crushable foam plasticity model is employed to analyze the scaffolds’ mechanical response under quasi-static compression. Experimental validation of the FE results demonstrates a favorable agreement, with an average percentage error of 12.27% ± 7.1%. Moreover, the yield strength and elastic modulus of the scaffolds are evaluated and compared, revealing notable differences between cubic and hexagonal closed-packed designs. The 30%, 50%, and 70% porous cubic pore-shaped bone scaffolds exhibit significantly higher yield strengths of 46.89%, 58.29%, and 66.09%, respectively, compared to the hexagonal closed-packed bone scaffolds at percentage strains of 5%, 6%, and 7%. Similarly, the elastic modulus of the 30%, 50%, and 70% porous cubic pore-shaped bone scaffolds is 42.68%, 59.70%, and 58.18% higher, respectively, than the hexagonal closed-packed bone scaffolds at the same percentage strain levels. Furthermore, it is observed in comparison with our previous study the μSLA-printed bone scaffolds demonstrate 1.5 times higher elastic moduli and yield strengths compared to the PJ-printed bone scaffolds.

## 1. Introduction

In the field of bone tissue engineering, the incorporation of biocompatible three-dimensional porous structures is often essential. These structures function as support and regeneration platforms [1]. When engineering synthetic bone scaffolds, the primary factors to consider are the architectural parameters, which must be carefully adjusted to promote effective bone regeneration while ensuring adequate mechanical strength [2]. Beam-based 3D-printed bone scaffolds demonstrate a tailored mechanical response characterized by a lattice structure composed of beams. This fabrication method allows for precise control over the internal architecture, resulting in optimized mechanical properties including strength and stiffness. Such scaffolds are well-suited for applications involving mechanical load bearing in bone tissue regeneration [3]. In contrast, TPMS (Triply Periodic Minimal Surfaces)-based 3D-printed bone scaffolds exhibit a distinctive mechanical response rooted in their intricate mathematical surfaces. These scaffolds leverage TPMS designs, known for their exceptional surface area and customizable porosities. This geometric complexity influences the scaffold’s mechanical behavior, making TPMS-based constructs highly promising for applications where specific mechanical responses, such as flexibility or compressibility, play a crucial role in advancing bone tissue engineering [4]. Conventional manufacturing techniques, including electrospinning, particle leaching, freeze drying, solvent casting, and gas foaming, can be employed to develop synthetic bone scaffolds. However, these techniques often exhibit limitations in terms of structural controllability and pore interconnectivity, which may adversely impact scaffold performance [5]. In contrast, additive manufacturing techniques are gaining prominence in the fabrication of synthetic bone scaffolds due to their ability to address the limitations of conventional techniques. Additive manufacturing provides unmatched flexibility for creating porous scaffolds with graded structures. This gradation can be achieved by systematically adjusting porosity, pore size, and strut thickness. While porosity and pore size influence mechanical performance and biological functionality, it is imperative to optimize wall or strut thickness to ensure ease of manufacturing [6]. Additive manufacturing enables precise control over architectural parameters, promotes suitable pore interconnectivity, and facilitates appropriate cell ingrowth capability, resulting in synthetic bone scaffolds with favorable mechanical strength and enhanced biological responses [7]. Similar to selective laser melting and fused deposition modeling, micro-stereolithography (μSLA) and PolyJet are additional additive manufacturing techniques that exhibit promising potential in advancing the development of synthetic bone scaffolds. These techniques allow for the precise definition of architectural parameters and compositions, contributing to the fabrication of scaffolds with highly controlled characteristics [8,9]. Moreover, it is possible to create mechanically robust synthetic scaffolds that possess a mechanical response closely resembling that of human bone tissues [10]. In a study by Maskery [11], the mechanical performance of lattice structures, manufactured using selective laser melting (SLM) with uniform and functionally graded properties, was predicted under quasi-static loading conditions. Choy [12] investigated the mechanical response of metal-based porous structures with functionally graded properties, which were developed using selective laser melting (SLM). Kadkhodapour [13] presented novel structure–property relationships for metal-based scaffolds. Lancae [14] performed a microstructural analysis using scanning electron microscopy on Ti6Al4V parts that were 3D-printed and exposed to a corrosive atmosphere. Furthermore, the influence of building direction on 3D-printed stainless steel parts with varying layer thicknesses was examined, and a comparison of the micro-hardness of parts fabricated using selective laser melting (SLM) was conducted [15,16]. Conversely, there has been comparatively less examination of the mechanical performance of polymer-based additively manufactured parts. [17]. The mechanical properties of various additively manufactured cellular structures, including polymers and composite materials such as foams and honeycombs, have been investigated by Zangana [18] and Gibson [19]. For evaluating the mechanical properties of lattice structures fabricated with polymer-based additive manufacturing, the two-step homogenization method introduced by Park [20] was utilized. Mahshid [21] conducted a study to explore the impact of architectural parameters on the compressive behavior of 3D-printed structures. Additionally, various approaches, including the FE method and geometric and mathematical models, were presented to predict the collapse strength of these structures. Moronia [22] and Kadkhodapour [23] investigated the influence of lattice design parameters on the dynamic properties and compression resistance of scaffolds fabricated using VeroBlue photopolymer resin, respectively. Numerous studies have employed FE to assess the architectural characteristics of synthetic bone scaffolds and examine their impact on mechanical properties, fluid transport, and cellular responses [24,25]. In a study by Smith et al. [26], FE models were utilized to compare computational results with compression test data on polymer-based porous structures fabricated using additive manufacturing techniques. The study concluded that analyzing the unit cells enables accurate prediction of the overall mechanical behavior of the developed structure. Nevertheless, there is currently a lack of numerical analysis examining the mechanical response of additively manufactured synthetic bone scaffolds under mechanical loadings using damage laws.

This study aims to investigate the damage behavior of 3D-printed polymeric bone scaffolds, which consist of cubic (C) and hexagonal closed-packed (H) pore shapes and have porosities of 30%, 50%, and 70%. FE modeling will be employed for this investigation. To depict the deformation of polymeric bone scaffolds, a crushable plasticity model is employed in the FE analysis. The utilization of a crushable foam plasticity model to analyze the 3D bone scaffolds’ deformation under quasi-static compression is performed in this study. The PolyJet (PJ) 3D printing technique is utilized to fabricate bone scaffolds using VeroClear material. Subsequently, the 3D-printed polymeric bone scaffolds undergo compression testing under quasi-static loading conditions to experimentally validate the FE study.

## 2. Materials and Methods

### 2.1. Finite Element Modelling

#### 2.1.1. Design of Polymeric Bone Scaffolds

**Figure 1:**
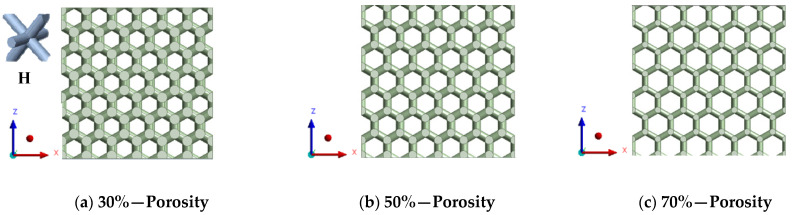

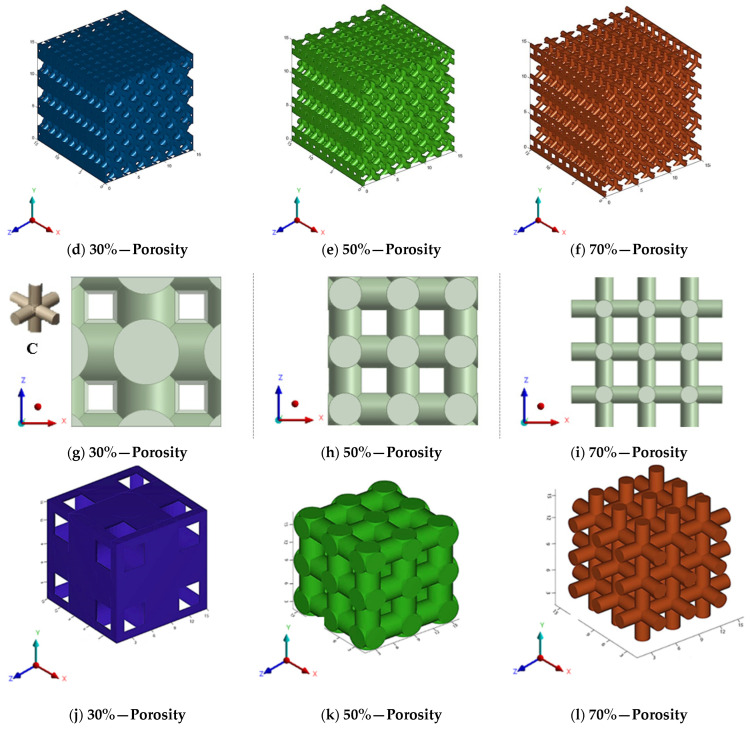
(**a**–**c**) The 2D views of the 3D CAD models with H unit cell; (**d**–**f**) the 3D views of the 3D CAD models with H unit cell; (**g**–**i**) the 2D views of the 3D CAD models with C unit cell; (**j**–**l**) the 3D views of the 3D CAD models with C unit cell.

This study involves the design of 3D FE models for bone scaffolds with porosities of 30%, 50%, and 70%. The overall dimensions of the models are 15 mm × 15 mm × 15 mm, and they feature a pore size of 2.5 mm. This study focused on the testing of materials and the characterization of its properties both experimentally and by modelling. For this reason, the current dimensions are applied in order to obtained higher resolution and more loading capacity for the principle proof and the validation of the mathematical model on wide range of data. PTC Creo 7.0 (Boston, Massachusetts, United States) is employed for the design process. To create the overall cubic structure of the bone scaffolds, cubic and hexagonal unit cells with 2.5 mm pore sizes and struts set at angles of 90° and 60°, respectively, are tessellated in 3D space. The strut diameters of the 3D FE models are adjusted to achieve the desired porosities of 30%, 50%, and 70%. These two are the most common and simple types used in the literature and their experimental response is well understood. The validation of these two common structures proves the efficacy of the FE model. For the hexagonal closed-packed and cubic FE models, the pore sizes are defined as the inscribed circle and length of sides, respectively. Figure 1 illustrates the detailed 3D FE models of the polymeric bone scaffolds from various viewpoints.

#### 2.1.2. Meshing

In FE analysis, the size of the mesh significantly impacts the FE results. Therefore, the initial investigation focused on examining the influence of mesh size on the mechanical response of polymeric bone scaffolds and, subsequently, on the damage. For this purpose, various sizes of tetrahedral meshes were generated on a 70% porous polymeric bone scaffold while keeping all other parameters constant. To measure convergence, the yield strength was calculated as an output parameter for each decreasing mesh size. Table 1 provides details regarding the element size, number of tetrahedral elements, computational time, and yield strength. The table demonstrates that reducing the element size from 5.0 mm to 0.8 mm led to a yield strength change of 7.04%. However, when further reducing the mesh size to 0.6 mm, the yield strength only changed by 0.80%. It is important to note that utilizing an element size of 0.6 mm required more computational power compared to the 0.8 mm element size. As a result, a tetrahedral mesh size of 0.6 mm was generated for each polymeric bone scaffold, as it provided FE results with good accuracy and reasonable computational time.

#### 2.1.3. Boundary Conditions

To conduct the FE simulations, three-dimensional computer-aided design (CAD) models of the polymeric bone scaffolds featuring cubic and hexagonal closed-packed unit cells were saved as .STEP files and imported into the explicit dynamic’s module of ANSYS 2020 R2. In order to replicate the clamps of a compression testing machine, plates were added at the top and bottom of the polymeric bone scaffolds.

**Figure 2:**
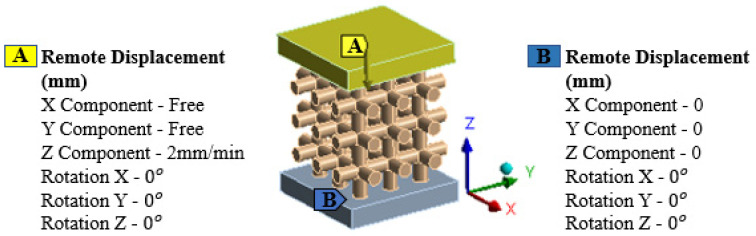
Top and bottom plates to mimic machine clamps, frictionless connection between plates and boundary conditions for FE simulations.

Frictionless connections were established between the polymeric bone scaffolds and the loading plates to mimic the realistic connection between the compression machine clamps and the as-built polymeric bone scaffolds. The top and bottom plates were defined as rigid bodies, while the polymeric bone scaffolds were defined as a flexible body. To simulate compression testing, remote displacement-controlled boundary conditions were applied to the plates. The polymeric bone scaffolds were quasi-statically compressed with a displacement rate of 2 mm/min. The bottom plate was fixed to the ground using the remote displacement, with all translation and rotation values set to zero. A compressive displacement was applied in the -z direction to the top plate of each polymeric bone scaffold to solve the FE problems. Figure 2 illustrates the FE simulation setup depicting the loading plates, connections, and boundary conditions.

#### 2.1.4. Crushable Foam Plasticity Model

**Figure 3:**
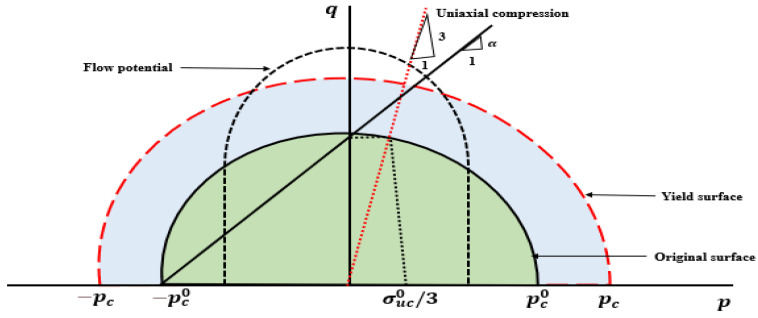
Crushable foam model: yield surface and flow potential in the p−q stress plane.

In order to analyze the deformation of the polymeric bone scaffolds, the crushable foam plasticity model was used in this study. The governing factors for the crushable foam model employing an isotropic hardening rule are the von Mises equivalent stress (q) and the hydrostatic pressure (p) [27]. In the stress plane of p−q, the yield surface is depicted as a centered ellipse at the origin shown in Figure 3.

Under the hydrostatic state, the yield surface expands along the pressure axis. The yield surface of the crushable foam model with isotropic hardening is defined as follows:

(1) $$F=\sqrt{q^{2}+\alpha^{2}p^{2}}-B$$

The expression involves *B*, which represents the q-axis dimension of the yield ellipse. Additionally, $\sigma_{\mathrm{uc}}$ denotes the uniaxial loading’s absolute compressive strength, while a signifies the shape factor of the yield ellipse, and their definitions are as follows:

(2) $$B=\alpha \times p_{c}=\sigma_{uc}\times \sqrt{1+\frac{a^{2}}{3}}$$

(3) α=3k9−k2

(4) k=σuc0pc0 

The parameters in above equations have specific meanings. α represents the shape of the yield ellipse in the p−q stress plane, while *B* denotes the size of the yield ellipse. Furthermore, $p_{c}$ signifies the yield strength under hydrostatic compression, k represents the compression yield stress ratio, $\sigma_{uc}^{0}$ stands for the initial yield strength under uniaxial compression, and $p_{c}^{0}$ represents the initial yield strength when subjected to hydrostatic compression. It is important to note that, due to the challenges associated with measuring hydrostatic compressive and tensile strength directly, several researchers [28] make assumptions regarding constant ratios k based on the experiments. Therefore, the sole parameter required to define the yield surface is the value of k. In the case of numerous low-density foams, the parameter α was found to be close to one, allowing the value of k to be set to unity [27] which corresponds to a value of 1. Moreover, flow potential is defined as [29];

(5) $$G=\sqrt{q^{2}+\beta^{2}p^{2}}$$

The parameter *β* represents the lengths of the principal axes of the flow potential ellipse in the p−q stress plane, and its correlation is determined by the plastic Poisson’s ratio.

(6) β=321−2vp1+vp

The geometry of the isotropic CF yield criterion in the q-p plane is determined by these relationships. Furthermore, the linear equation below was employed to establish the evolving yield stress’s work hardening slope (*H*) [29];

(7) H=(σeσ^)×hσ+( 1−σeσ^ )×hp

$\sigma_{e}$ represents the von Mises effective stress while $\hat{\sigma }$ denotes the equivalent stress. Additionally, $h_{\sigma }$ and $h_{p}$ indicate the slopes of the stress versus logarithmic plastic strain curve during uniaxial and hydrostatic compression, respectively. Several FE solvers are integrated with the crushable foam plasticity model which requires five parameters for its complete definition. These parameters include the modulus of elasticity, Poisson’s ratio, density, stress–strain curve, and maximum tensile stress for tension cut-off. The values of these parameters, as listed in Table 2, were retrieved from our previous study [8]. The previous study focused on investigating the mechanical response of 3D-printed standard solid samples under compression. For the FE analysis in this study, the stress–strain curves obtained from the 3D-printed standard solid samples in the z-direction were used and presented in Section 3.1. This choice was made because the polymeric bone scaffolds utilized in this study were printed in the same z-direction.

### 2.2. Experimental Setup

#### 2.2.1. Development of Polymeric Bone Scaffolds and Solid Samples

To facilitate the fabrication process, the 3D CAD models of the polymeric bone scaffolds were initially converted into the widely used .stl format. Subsequently, a PolyJet printer was employed to print the polymeric bone scaffolds using commercially available materials. The Stratasys Objet260 Connex 1 (Stratasys, EMEA Regional Office (Baden-Baden, Germany)) PolyJet printer is equipped with a high-capacity material cabinet capable of holding up to eight sealed 3.6 kg cartridges. This enables the loading of three different model materials simultaneously and facilitates hot-swapping when necessary. The printer’s net build size is 255 × 252 × 200 mm^3^ (10.0 × 9.9 × 7.9 in.). The printer provides exceptionally accurate printing by providing horizontal build layers with a maximum thickness of 16 microns (0.0006 in.). The build resolution is equally impressive with a 600-dpi resolution for both the *x* and *y* axes and an outstanding 1600 dpi resolution for the z-axis. The printer delivers enhanced precision, with features smaller than 50 mm falling within a range of 20–85 microns and full model sizes up to 200 microns.

**Figure 4:**
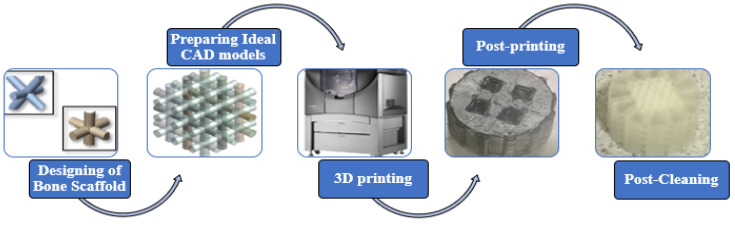
Summary of stages involved in the development of additively manufactured polymeric bone scaffolds and standard solid samples.

**Figure 5:**
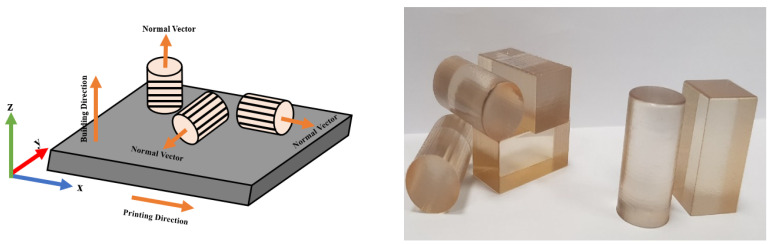
Standard solid samples drawn in x-, y-, and z-, directions and 3D-printed in the z-direction. Lines represent the build layers’ direction [8].

VeroClear from STRATASYS was the specific material used for the printing. In this study on bone scaffold development using PolyJet 3D printing, VeroClear is selected due to its initial advantages. Its transparency aids in visualizing the porosity and scaffold structure during the design and prototyping phases. Additionally, VeroClear is a popular material for 3D printing substrates in biomedical engineering applications [30,31,32], which makes it a suitable choice for our study. Its cost-effectiveness during the early stages allowed us to focus on porosity and deformation behavior analysis, with biocompatibility enhancements planned for future research phases. The PolyJet printer required approximately ninety minutes to print six polymeric bone scaffolds. After printing, the support material (SUP706) was removed using pressurized water, followed by the elimination of residual particles using compressed air. The support material was a non-toxic gel-like photopolymer support, manufactured and designed by Stratasys (North America—Stratasys Units). During the PolyJet printing process, SUP 706 was simultaneously deposited alongside the model material, but in areas where support was needed. These support structures were used to uphold the overhanging features and complex geometries of the model. On the other hand, in our previous study [8], in μSLA (Krämpferstraße 4, 99084 Erfurt, Germany), each scaffold took approximately three hours and twelve minutes to print individually, with a thickness of 0.025 mm. The support material of the as-built polymeric bone scaffolds was removed via sonication in isopropyl alcohol (IPA) for twenty minutes, and the solid support beams were manually removed [8]. The IPA was obtained from Sigma Aldrich, Ireland, and has been used for the rinsing of 3D-printed parts by the co-authors previously [33]. Figure 4 provides a visual representation of the stages involved in the development of the polymeric bone scaffolds. These same stages were followed for fabricating the standard solid samples required for the crushable foam plasticity model in FE modeling. For the solid samples, three replicates were created in the x-, y-, and z-directions and printed in the x-direction using the printer depicted in Figure 5. In Figure 5, the crooked appearance of the samples is attributed to the angular perspective from which the images were captured. It should be noted that the build direction for the polymeric bone scaffolds was in the z-direction. The different printing directions were utilized to assess the effect of the printing direction on the mechanical properties of the samples and to determine the most relevant results for the crushable foam plasticity model. A comprehensive discussion on the experimental investigation of the 3D-printed solid samples can be found in our previous study [8].

#### 2.2.2. Structural Characterization of Polymeric Bone Scaffolds

Following the 3D printing process, the Keyence-Digital microscope VHX-2000 (Osaka, Japan) was utilized to capture optical microscopic images of the as-built polymeric bone scaffolds. The purpose was to identify any variations in the architectural measurements of the as-built scaffolds compared to the CAD-based models, as these differences could potentially explain the disparities between the experimental and FE mechanical responses of the scaffolds. To capture the images, the Keyence-Digital microscope VHX-2000 employed a progressive scanning method at a rate of 28 frames per second, with a resolution of 8 million pixels. Prior to imaging, the x-y motorized stage was initialized, and adjustments were made to the color and brightness settings. Subsequently, the x-y motorized stage was moved and tilted to ensure optimal angles for capturing high-quality images of the polymeric bone scaffolds.

#### 2.2.3. Quasi-Static Compression Testing

**Figure 6:**
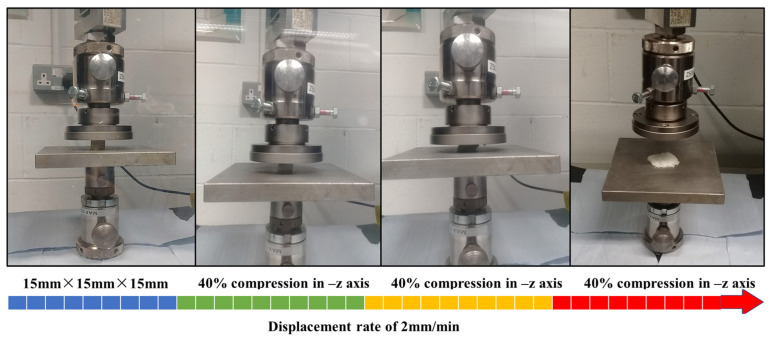
Different levels of compression testing of additively manufactured polymeric bone scaffolds and 3D-printed standard solid samples.

After the optical microscopy imaging, the as-built polymeric bone scaffolds were subjected to compression testing using the Zwick/Roel Z50 (Zwick/Roell GmbH & Co. KG, Ulm, Germany) universal testing machine, which was integrated with the Zwick TestXpert III simulation software. The characterization of the as-built scaffolds followed the ASTM D-695 standard, employing a deformation rate of 2 mm/min and a maximum loading capacity of 50 KN. To ensure proper contact between the mating parts and minimize potential sliding effects, a pre-loading value of 5 N was applied. In this study, the struts are acting as short columns because the slenderness ratio is less than 9. Short columns fail due to compression instead of buckling, which eliminates the need for bucking supports during compression testing. The total samples were 28 and in order to determine the 95% confidence level, three repetitions of compression tests on each sample were performed. Therefore, a total of 84 samples (three copies of each sample) were printed to perform compression tests. The compression test was repeated three times for each of the three replicates of the as-built polymeric bone scaffolds. Figure 6 illustrates the different stages of compression. The force versus displacement curves obtained from Zwick TestXpert III were used to construct stress–strain diagrams for the as-built polymeric bone scaffolds. A similar procedure was employed to characterize the mechanical behavior of the 3D-printed standard solid samples [8]. The mechanical properties derived from the compression testing of the 3D-printed solid samples were subsequently utilized in the FE analysis conducted in this study.

## 3. Results

### 3.1. Experimental Validation of FE Results

**Figure 7:**
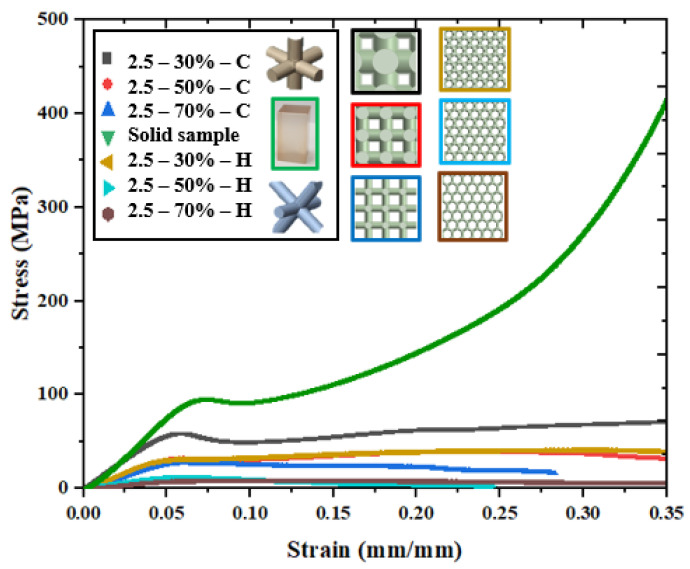
Experimental stress–strain curves for 3D-printed porous polymeric bone scaffolds and 3D-printed standard solid samples.

Figure 7 shows the mechanical behavior of polymeric bone scaffolds with porosities of 30%, 50%, and 70%, and a pore size of 2.5 mm, for experimental validation. The individual stress–strain curve in Figure 7 is the average of the three replicates of polymeric bone scaffold with an average percentage error of less than 1% ± 0.32). The inclusion of the solid sample curve in Figure 7 serves as a point of comparison for the porous samples (30%, 50%, and 70% C). By showing the solid sample curve, the author highlighted the impact of the varying porosities (30%, 50%, and 70% C) on the material’s properties.

The stress–strain curves for all the bone scaffolds exhibit a similar trend to that of cellular materials. Following the elastic domain, energy absorption takes place during the plateau region until the onset of densification. In cellular materials, strain hardening or strain softening can occur at the beginning of the plateau region. In this study, for the 30% and 50% porous polymeric bone scaffolds with a cubic unit cell, a stress decline was observed at the end of the first peak of the stress–strain curve, indicating strain softening leading up to the beginning of densification. Conversely, no densification region was observed for the 70% porous polymeric bone scaffolds with a cubic unit cell. Among the hexagonal closed-packed unit cell types, only the 30% porous bone scaffolds exhibited a plateau region and densification region. Densification was not observed in any other bone scaffolds. The presence of a densification region is dependent on the deformation and failure mechanisms that occur during the crushing stage, particularly at higher strain values. The absence of a densification region and subsequent terminal hardening were anticipated in cases where deformation involved the highly brittle failure of thin struts and the delamination of the material. Furthermore, the stress–strain curves revealed that the 50% porous bone scaffolds with a hexagonal pore shape and the 70% porous bone scaffolds with a cubic pore shape did not approach the densification region up to a strain value of 0.35 mm/mm. This behavior aligns with the findings of our previous study [8], which examined bone scaffolds fabricated using the μSLA technique. In Figure 6, a comparison is also presented between the mechanical responses of the 3D-printed standard solid sample, cubic polymeric bone scaffolds, and hexagonal polymeric bone scaffolds. It can be observed that the elastic moduli and yield strength values are higher for the cubic pore shape compared to the hexagonal pore shape. Additionally, the mechanical response of denser or less porous polymeric bone scaffolds closely resembles that of the solid sample, whereas it deviates in the case of higher porosity polymeric bone scaffolds.

**Figure 8:**
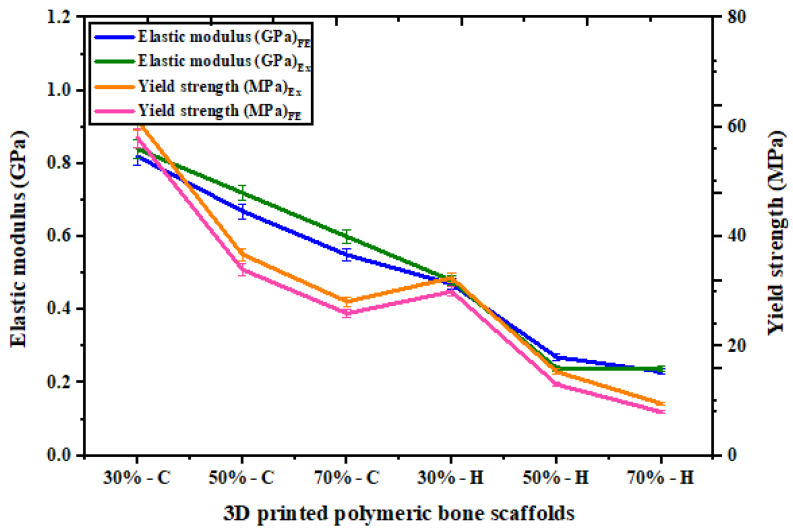
Experimental and FE elastic moduli and yield strengths of polymeric bone scaffolds.

The mechanical properties of the polymeric bone scaffolds, as determined by the crushable foam plasticity model, showed good agreement with the experimental data, with an average percentage error of 12.27% ± 3.05) (% error = (measured value − numerical value)/measured value)). A comparison between different regions of the experimental and FE stress–strain behavior is presented in Section 3.2. The elastic regions of the experimental and FE stress–strain curves exhibit good agreement with each other. In the plastic region, although the magnitude of the FE stress was higher than the experimental stress, the trend of the plateau stress matched well with the numerical results. Furthermore, the Young’s moduli and yield strengths of the polymeric bone scaffolds, as shown in Figure 8, demonstrate that the crushable foam plasticity model accurately predicts the maximum and plateau stress with an overall percentage error of 12.27% when compared to the experimental values. A similar pattern is observed for bone scaffolds fabricated through μSLA, with a percentage error of less than 3% [8].

**Figure 9:**
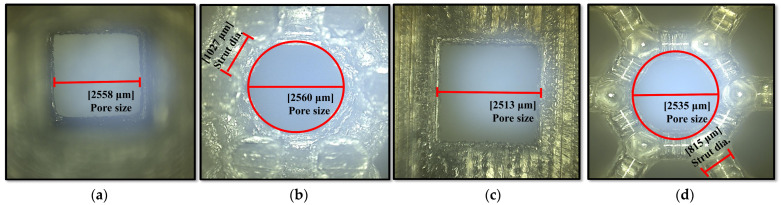
Optically measured architectural parameters of as-built polymeric bone scaffolds using a Keyence-Digital microscope VHX-2000: (**a**) cubic pore shape with 30% porosity, (**b**) hexagonal pore shape with 30% porosity, (**c**) cubic pore shape with 50% porosity, and (**d**) hexagonal pore shape with 50% porosity [8].

The disparity between the experimental and FE mechanical properties of the polymeric bone scaffolds can be attributed to notable variations in strut diameters and deviations of the struts from the building direction. Figure 9 presents microscopic images of the as-built polymeric bone scaffolds, which were captured using the Keyence-Digital microscope VHX-2000, to investigate these geometric deviations.

The variations in the architectural parameters between the as-built polymeric bone scaffolds and the CAD-based models (actual) are summarized in Table 3. Upon geometric characterization, it was observed that the strut diameter of the as-built polymeric bone scaffolds gradually increased as the porosity decreased, with an average difference of approximately 2.53% and 2.54%, respectively. The increase in strut diameters in each as-built polymeric bone scaffold was likely due to the overcuring of the printed layers during the fabrication process. Similarly, a deviation of approximately 2.5% was observed in the architectural parameters between the as-built polymeric bone scaffold and the CAD-based polymeric bone scaffolds in the case of μSLA [8].

### 3.2. Deformation in Polymeric Bone Scaffolds

**Figure 10:**
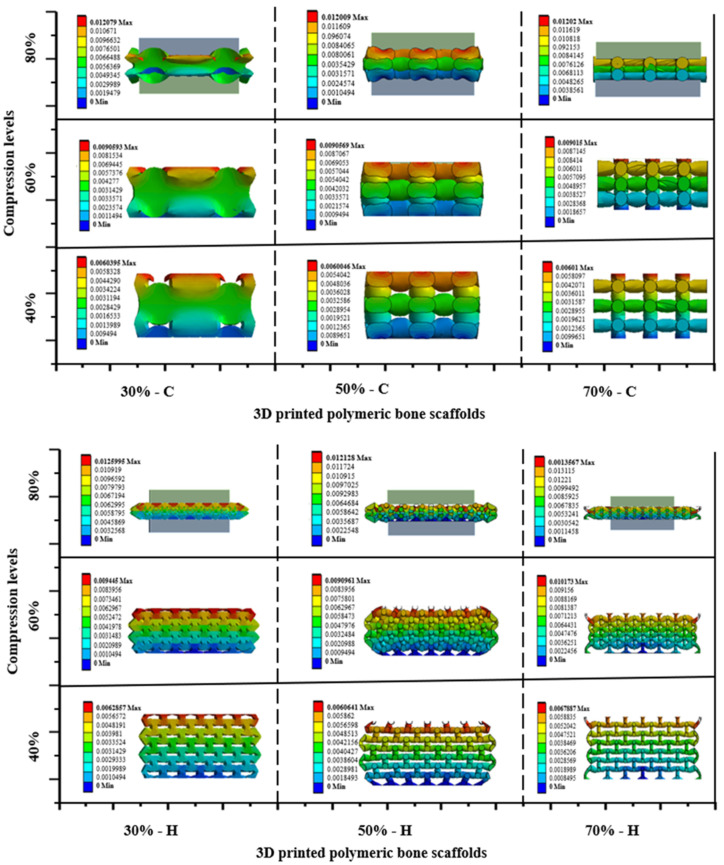
Deformation of polymeric bone scaffolds under the compression levels of 40%, 60%, and 80%.

The numerical analysis of the deformation of polymeric bone scaffolds under compression levels of 40%, 60%, and 80% is depicted in Figure 10. It is observed that the 30% porous polymeric bone scaffolds with a hexagonal pore shape exhibit deformations approximately 4% higher than those of the polymeric bone scaffolds with a cubic pore shape. The percentage error decreases to 1% as the porosity increases from 50% to 70%. Overall, the polymeric bone scaffolds consistently displayed outward bulging during compression, progressing from 40% to 80%.

**Figure 11:**
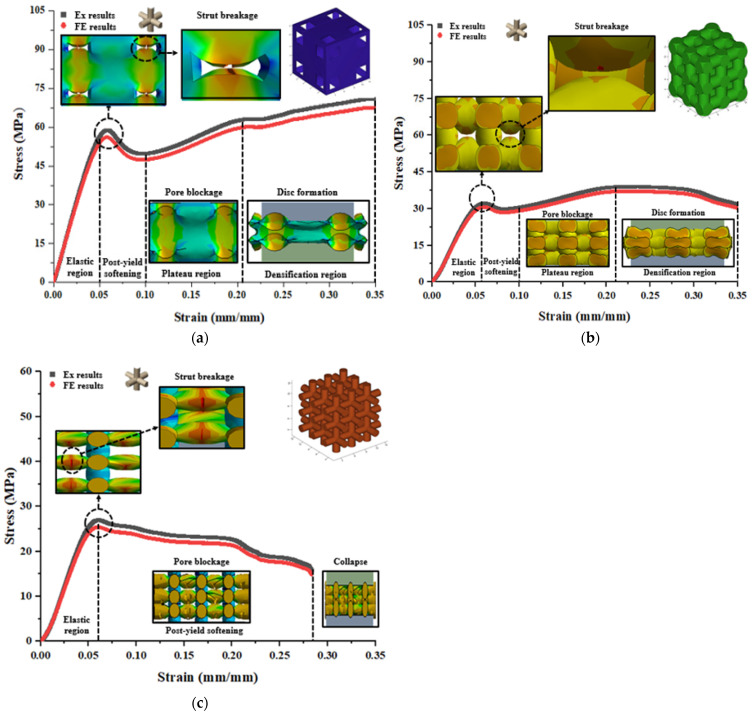
FE deformation mechanism through compressive stress contours of cubic pore shape at different level of strains which consisted of (**a**) 30%, (**b**) 50%, and (**c**) 70% porosity in comparison with the actual deformation behavior of polymeric bone scaffolds.

In a similar manner, compressive stress contours were extracted to investigate the stress behavior of polymeric bone scaffolds with a cubic pore shape after conducting FE analysis, as illustrated in Figure 11. Figure 11a demonstrates the uniform deformation of vertical struts in the elastic region for the 30% porous polymeric bone scaffolds. Following the first peak of maximum stress in the elastic region, post-yield softening occurred, leading to the initiation of strut breakage. Subsequently, deformation was accompanied by pore blockage due to extensive strut failure in the plateau region, extending until the beginning of the densification region. Eventually, the entire structure was crushed and transformed into a disc-like shape at the end of densification. A similar failure pattern was observed for the 50% porous polymeric bone scaffolds, as shown in Figure 11b. However, in the case of the 70% porous polymeric bone scaffolds depicted in Figure 11c, post-yield softening was eliminated, and the collapse of the structure occurred before reaching a strain of 0.35 (mm/mm) due to continuous buckling and breakage of micro-struts. Minor stress fluctuations were also observed during the failure process, corresponding to the failure and buckling of micro-struts in the plateau and densification regions.

**Figure 12:**
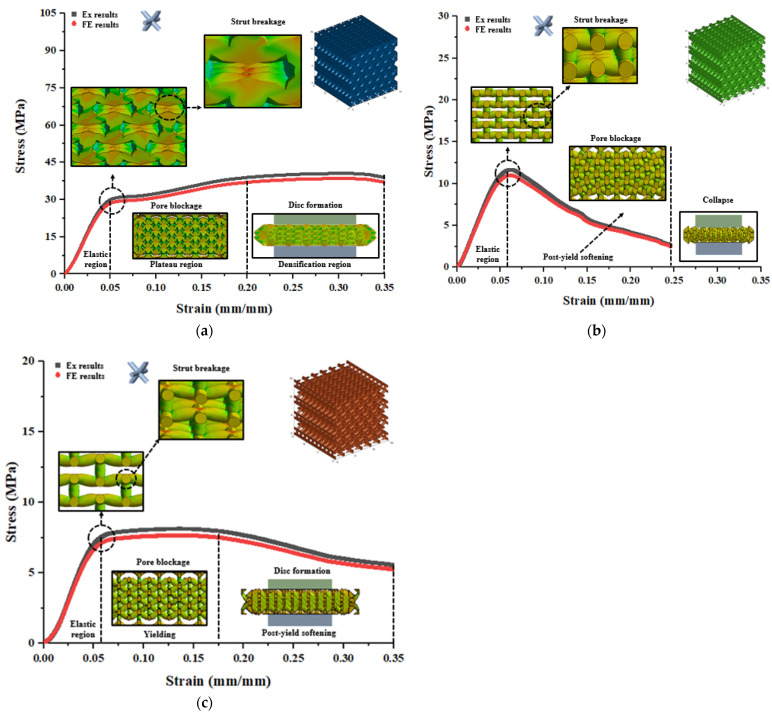
FE deformation mechanism through compressive stress contours of hexagonal closed-packed pore shape at different level of strains which consisted of (**a**) 30%, (**b**) 50%, and (**c**) 70% porosity in comparison with the actual deformation behavior of polymeric bone scaffolds.

Polymeric bone scaffolds with a hexagonal closed-packed pore shape, featuring porosities of 30%, 50%, and 70%, exhibited distinct mechanical responses. Compressive stress contours obtained from the FE analysis were assigned to different regions based on their corresponding strain, as depicted in Figure 12.

Figure 12a displays the deformation behavior of the 30% porous bone scaffolds, characterized by uniform deformation accompanied by strut breakage in the elastic region. Subsequently, deformation progresses with pore blockage were observed in the plateau region until the beginning of the densification region. Eventually, the entire structure underwent crushing and transformed into a disc-like shape at the end of the densification. In Figure 12b, the deformation is followed by post-yield softening, resulting in the collapse of the structure before reaching a strain of 0.35 (mm/mm). Similarly to the 30% porous bone scaffolds, the deformation of the 70% porous bone scaffolds, shown in Figure 12c, is accompanied by pore blockage in the plateau region until the beginning of the densification region. Subsequently, the entire structure experiences crushing and transforms into a disc-like shape at the end of densification.

## 4. Discussion

The fabrication of bone scaffolds with complex shapes and precise architectural parameters, as well as appropriate mechanical properties, is crucial in bone tissue engineering applications for achieving the accurate mimicry of native tissue [34]. Additive manufacturing techniques offer control over scaffold fabrication, enabling the creation of biocompatible scaffolds with suitable mechanical properties that can serve as bone substitutes in orthopedics [35]. FE analysis has gained popularity in tissue engineering as a means to enhance the design of bone scaffolds by investigating the influence of architectural parameters on their mechanical and fluid transport properties [26]. Despite a limited number of studies on the deformation and failure mechanisms of additively manufactured bone scaffolds, accurately predicting their deformation and failure mechanisms remains a significant challenge. Ongoing research aims to develop material models that can precisely simulate the deformation of bone scaffolds and improve our understanding of their behavior. With the aim of investigating the deformation of polymeric bone scaffolds, the authors conducted FE analysis. In this study, six CAD-based polymeric bone scaffolds were designed and fabricated using the PolyJet (PJ) method. To define the plastic range of the polymeric bone scaffolds, a crushable foam plasticity model was utilized in the FE modeling. Compression testing was performed on both standard solid samples and polymeric bone scaffolds to experimentally validate the results obtained from the FE analysis. The observed discrepancy of 12.27% between the experimental data and FE results may be attributed to assumptions of material isotropy and deviations of approximately 2.5% in the architectural parameters between the CAD-based polymeric bone scaffolds and the as-built polymeric bone scaffolds. The strut diameters in nearly all of the as-built polymeric bone scaffolds were found to be larger compared to the CAD-based polymeric bone scaffolds, likely due to the overcuring of layers during the printing process. Additionally, the deviations in architectural parameters led to a reduction in the porosity of the as-built polymeric bone scaffolds, resulting in porosity values of approximately 29.29%, 49.41%, and 69.72%, which closely matched those of the CAD-based polymeric bone scaffolds. This indicates the high fidelity of the CAD-based bone scaffold models used in additive manufacturing. Therefore, it can be expected that CAD-based FE models provide a reasonably accurate representation of the actual porous bone scaffolds. However, there were some deviations in the architectural parameters of the as-built bone scaffolds compared to the nominal values, which could potentially impact the FE results. To address this, it is suggested that the as-built bone scaffolds be reconstructed using optical microscopy images, as this approach can help minimize discrepancies between experimental and FE results in future studies and enhance the accuracy of the FE analysis. In this study, CAD-based polymeric bone scaffolds were designed with various combinations of architectural parameters, and their deformation patterns were investigated using FE analysis with a damage model. The results revealed distinct deformation behaviors for different types of scaffolds. For the polymeric bone scaffolds with a cubic pore shape and porosities of 30% and 50%, a stress fall was observed after the initial peak of the stress–strain curve, indicating a softening region followed by strut failure. The plateau region showed pore blockage leading to the beginning of the densification region. However, in the case of the 70% porous polymeric bone scaffolds with the same pore shape, no densification region was observed. This absence of densification was anticipated due to the brittle failure of thin struts during deformation. Regarding the polymeric bone scaffolds with a hexagonal closed-packed pore shape, only the 30% porous scaffolds exhibited a plateau and densification region. The 50% and 70% porous scaffolds experienced crushing before reaching the densification region, up to a strain value of 0.35 mm/mm. Comparing the two pore shapes, it was noted that although the mechanical response of the cubic pore shape was higher than that of the hexagonal closed-packed pore shape, the failure in the latter was more uniform. The mechanical properties of the 50% porous scaffolds, irrespective of pore shape, closely resembled those of human bone. Additionally, the crushable foam plasticity model proved effective in simulating the deformation of polymeric bone scaffolds, offering valuable insights for redesigning scaffolds to prevent damage to native tissues in bone tissue engineering applications.

## 5. Conclusions

This study aimed to evaluate the crushable foam plasticity model’s effectiveness in predicting the deformation and failure mechanisms of 3D-printed polymeric bone scaffolds through FE analysis. Experimental data revealed a discrepancy, indicating that the crushable foam plasticity model is capable of estimating the maximum and plateau stresses of polymeric bone scaffolds. To enhance understanding of the deformation of 3D-printed polymeric bone scaffolds, it is necessary to develop more detailed numerical methods that incorporate failure modes. This knowledge can then be used to redesign and develop new structures that are more suitable for bone replacement, taking into account the observed modes of failure and deformation patterns.

## Figures and Tables

**Table 1:** Tetrahedral entities with computational time and yield strength for convergence.

	Mesh 1	Mesh 2	Mesh 3
Polymeric bone scaffold	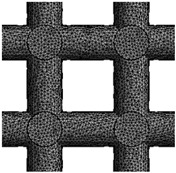	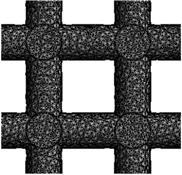	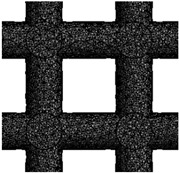
Element size (mm)	5.0	3.0	2.0
No. of tetrahedral elements	333,945	656,999	1,121,900
Computational time (min)	15	20	28
Yield strength (MPA)	26.57	25.54	25.47
	**Mesh 4**	**Mesh 5**	**Mesh 6**
Polymeric bone scaffold	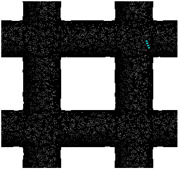	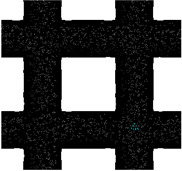	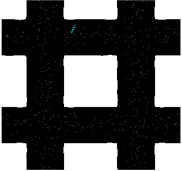
Element size (mm)	1.0	0.8	0.6
No. of tetrahedral elements	3,198,744	4,550,711	7,472,326
Computational time (min)	41	47	72
Yield strength (MPA)	25.38	24.90	24.70

**Table 2:** Material properties for the crushable foam plasticity model used in FE modelling [8].

Parameters	Values
Elastic modulus (GPa)	1.6
Poison ratio	0.32
Maximum tensile stress (MPa)	50
Density (kg/m^3^)	1190

**Table 3:** Deviations in the architectural parameters of CAD-based (actual) polymeric bone scaffolds and additively manufactured polymeric bone scaffolds.

Pore Shapes	Porosities (%)		Diff. (%)	Pore Sizes (mm)		Diff. (%)	Strut Diameters (mm)		Diff. (%)
	Actual	As-Built		Actual	As-Built		Actual	As-Built	
H	30	29.29 ± 4.05%	2.37	2.50	2.56 ± 5.75%	2.34	1.004	1.027 ± 1.08%	2.34
H	50	49.22 ± 3.42%	1.56	2.50	2.54 ± 4.05%	1.57	0.802	0.815 ± 0.09%	1.57
H	70	69.45 ± 3.15%	0.78	2.50	2.52 ± 4.05%	0.79	0.590	0.595 ± 0.08%	0.78
C	30	29.29 ± 1.33%	2.37	2.50	2.56 ± 4.05%	2.34	6.600	6.754 ± 0.13%	2.35
C	50	49.41 ± 1.05%	1.18	2.50	2.53 ± 4.05%	1.18	3.280	3.319 ± 0.11%	1.18
C	70	69.72 ± 0.08%	0.40	2.50	2.51 ± 4.05%	0.40	1.750	1.757 ± 0.09%	0.41

## Data Availability

The data presented in this study are available on request from the corresponding author.
